# Influence of renal function on stroke outcome after mechanical thrombectomy: a prospective cohort study

**DOI:** 10.1186/s12883-020-01720-5

**Published:** 2020-04-14

**Authors:** Xiding Pan, Feng Zhou, Rui Shen, Yubing Zhu, Hisatomi Arima, Jie Yang, Junshan Zhou

**Affiliations:** 1 Department of Pharmacy, Nanjing First Hospital, Nanjing Medical University, Nanjing, China; 2 Department of Neurology, Nanjing First Hospital, Nanjing Medical University, Nanjing, China; 3 Department of Preventive Medicine and Public Health, Faculty of Medicine, Fukuoka University, Fukuoka, Japan; 4 Department of Neurology, The First Affiliated Hospital of Chengdu Medical College, Chengdu, 610500 China

**Keywords:** Estimated glomerular filtration, Mechanical thrombectomy, Outcomes, Stroke

## Abstract

**Background:**

For acute ischemic stroke (AIS) patient receiving mechanical thrombectomy (MT), renal dysfunction was an independent risk factor of contrast-induced nephropathy which may affect clinical outcomes. However, the influence of renal function on stroke outcomes is still controversial. Thus, we aim to investigate the association between renal function and outcomes of AIS patients receiving MT.

**Methods:**

All consecutive stroke patients receiving MT were included in a prospective stroke registry in China from April 2015 to February 2019. Estimated glomerular filtration rate (eGFR) was measured on admission and categorized into G1 (≥ 90 ml/min/1.73 m^2^), G2 (60–89 ml/min/1.73 m^2^), G3a (45–59 ml/min/1.73 m^2^) and G3b-5 (≤44 ml/min/1.73 m^2^). Multivariable logistic regression analysis was performed to evaluate the association between eGFR and recanalization rate (thrombolysis in cerebral infarction scale 2b-3), symptomatic intracranial hemorrhage (sICH), death in hospital, death at 3 months and poor functional outcome (modified Rankin Scale 3–6 at 3 months).

**Results:**

A total of 373 patients were included in the study. Of them, 130 (34.9%) patients were in the eGFR group G1, 170 (45.6%) in G2, 46 (12.3%) in G3a, 27 (7.2%) in G3b–5. In multivariable logistic regression analysis, reduced eGFR was associated with increased risk of sICH (G3a, *p* = 0.016) and 3-month death (G3b–5, *p* = 0.025). However, no significant effects were observed between reduced eGFR and the risk of recanalization rate (*p* = 0.855), death in hospital (*p* = 0.970), and poor functional outcome (*p* = 0.644).

**Conclusions:**

For AIS patients underwent MT, reduced eGFR was associated with increased risk of sICH and 3-month death. However, there were no appreciable effects of reduced eGFR on recanalization rate, death in hospital and 3-month functional outcome.

## Background

Chronic kidney disease (CKD) is an increasing global public health issue. Worldwide, the prevalence of CKD is estimated to be 8–16% [1]. In China, it constitutes 4.5% of all hospitalizations [2].

CKD is an independent risk factor of cardiovascular disease [3]. For stroke patients, some studies suggested that CKD with decreased estimated glomerular filtration rate (eGFR) significantly increased the risk of stroke mortality [4, 5]. In intravenous thrombolysis (IVT) therapy, one study with large sample size revealed that low GFR was independently associated with poor outcome and the risk of symptomatic intracerebral hemorrhage (sICH) [6]. However, the results of other studies were conflicting [7, 8].

Besides IVT, mechanical thrombectomy (MT) recently has been proven to be an effective therapy for acute ischemic stroke (AIS) [9]. In patients with 6 to 24 h after onset, MT is the only reperfusion strategy for the selected patients [10]. However, during the MT procedure, over 90% iodinated contrast media (CM) is eliminated by the kidney [11]. Renal dysfunction can cause the accumulation of CM in blood plasma and increase its nephrotoxicity. Thus, compared with other AIS treatment, in MT procedure, patients with reduced eGFR are at a higher risk of acute kidney injury (AKI), which increases the hospital mortality [12, 13]. Up to present, the relationship between renal function and outcomes after MT has not been sufficiently evaluated.

In this study, we aimed to investigate the influence of renal function on outcomes of MT and therefore provide information to improve patient selection for MT.

## Methods

### Study design and patients

Nanjing First Hospital Stroke Registry is a prospective cohort study. It was designed to achieve comprehensive information of stroke patients in Nanjing, China. The study protocol was approved by the hospital ethics committee. Form April 2015 to February 2019, all consecutive AIS patients receiving MT were included in the study. The patient’s inclusion criteria for MT were in accordance with national guideline recommendation and neuro-interventionist’s evaluation. The patients also received subsequent standard treatment for AIS. Written informed consent was obtained from each patient or their statutory.

### Data collection

On admission, baseline characteristics were recorded including demographic characteristics, past medical history, disease severity evaluation, vital signs and crucial laboratory tests [glucose, platelet count, serum creatinine, prothrombin time and international normalized ratio (PT/INR)]. Other laboratory tests (lipid panel, liver function panel) were obtained during hospitalization. Magnetic resonance imaging (MRI) was performed before the MT procedure. Computerized tomographic scan (CT) was performed at 24 h after MT or anytime if neurological deterioration was observed. At 3 months after discharge, patients were evaluated by the telephone follow-up.

### Renal function

Serum creatinine was measured on admission. eGFR was evaluated by the CKD Epidemiology Collaboration (CKD-EPI) equation [14]. According to the guideline [15], renal function was categorized into G1 (≥ 90 ml/min/1.73 m^2^), G2 (60–89 ml/min/1.73 m^2^), G3a (45–59 ml/min/1.73 m^2^), G3b (33–44 ml/min/1.73 m^2^), G4 (15–29 ml/min/1.73 m^2^) and G5 (< 15 ml/min/1.73 m^2^). reduced eGFR was defined as GFR < 90 ml/min/1.73 m^2^ (G2–5). Renal dysfunction was defined as GFR < 60 ml/min/1.73 m^2^ (G3a-5). eGFR ≥90 ml/min/1.73 m^2^ (G1) was the reference group, eGFR ≤44 ml/min/1.73 m^2^ (G3b-5) were pooled together due to the small sample size.

### Outcome assessment

The outcome measures were (1) recanalization rate, defined as thrombolysis in cerebral infarction scale (TICI) 2b–3; (2) sICH, defined as hemorrhage on CT scan with neurological deterioration of ≥4 points on National Institutes of Health Stroke Scale (NIHSS) score [16]; (3) death in hospital; (4) death at 3 months; (5) long-term functional outcome, measured by modified Rankin Scale (mRS) at 3 months. Poor functional outcome was defined as mRS 3–6.

### Statistical analysis

Continuous variables were presented as mean with standard deviation (SD) or median with inter-quartile range (IQR) when appropriate. Categorical variables were presented as numbers with percentages. First, we compared baseline characteristics across the 4 eGFR groups. We used flowing methods: for continuous variables, we used one-way ANOVA test or Kruskal–Wallis test; for categorical variables, we used chi-square test or Fisher’s exact test. Then we conducted an univariable analysis using binary logistic regression models to evaluate the association between each clinical characteristic and each clinical outcome, in which eGFR was presented as a continuous variable. In multivariable logistic regression analysis, eGFR was categorized into 4 groups [G1 (reference group), G2, G3a, and G3b-5]. The following covariates were adjusted in the multivariable models: (1) variables with *p* < 0.1 in the univariable analysis (2) factors identified by previous studies which are clinically relevant to the outcomes. We performed two models: model 1 was adjusted for age and sex. Model 2 was adjusted for all the potential confounders. SPSS (version 22.0 for Windows; IBM Inc., Armonk, NY, USA) was used for statistical analysis. *p* < 0.05 was considered significant.

## Results

### Baseline clinical characteristics

A total of 374 AIS patients received MT treatment from April 2015 to February 2019, 1 were excluded due to lack of serum creatinine value (*n* = 373). At 3 months, 15 patients were lost to follow-up. Among eligible patients, 130 (34.9%) patients were in the eGFR group G1 (≥ 90 ml/min/1.73 m^2^), 170 (45.6%) were in G2 (60–89 ml/min/1.73 m^2^), 46 (12.3%) were in G3a (45–59 ml/min/1.73 m^2^) and 27 (7.2%) were in G3b–5 (≤44 ml/min/1.73 m^2^). Baseline characteristics are presented in Table 1. Patients with reduced eGFR were older and more commonly had hypertension, coronary artery disease, atrial fibrillation, previous TIA/stroke and higher NIHSS score. They were more likely to have higher PT/INR and glucose level. They also had lower total cholesterol and LDL levels. In addition, our study showed that patients with low GFR were more prone to have cardioembolism as stroke etiology (*p* = 0.013) whilst patients with higher GFR were large artery atherosclerosis (*p* = 0.047) (Table 1).

**Table 1:** Baseline characteristics of patients according to eGRF

	eGFR (ml/min/1.73 m^2^)				
	≤44 (G3b–5) *n* = 27	45–59 (G3a) *n* = 46	60–89 (G2) *n* = 170	≥90 (G1) *n* = 130	*p*
**Demographic**					
Age, y	82 (75–85)	77 (66–83.3)	76 (70–81)	63.5 (55–70)	**< 0.001**
Male	16 (59.3)	30 (65.2)	102 (60.0)	87 (66.9)	0.621
Weight, Kg	64 (60–70)	65 (60–70)	65 (60–70)	65 (60–75)	0.633
**Cardiovascular risk factors**					
Hypertension	19 (70.4)	39 (84.8)	131 (77.1)	82 (63.1)	**0.011**
Diabetes	8 (29.6)	10 (21.7)	32 (18.8)	24 (18.5)	0.573
Coronary artery disease	8 (29.6)	15 (32.6)	46 (27.1)	15 (11.5)	**0.002**
Atrial fibrillation	14 (51.9)	19 (41.3)	69 (40.6)	26 (20.0)	**< 0.001**
TIA/stroke	5 (18.5)	14 (30.4)	51 (30.2)	22 (16.9)	**0.04**
Smoking^a^	6 (22.2)	19 (41.3)	50 (29.6)	57 (43.8)	**0.026**
**Clinical features on admission**					
NIHSS score	16 (14–20)	16.5 (11–24)	16 (12–20)	13.5 (9–18)	**0.005**
OTT, min	202 (134–345)	178 (136–309)	195 (130–333)	211 (155–379)	0.352
SBP, mmHg	146 ± 22	144 ± 21	144 ± 24	138 ± 23	0.127
DBP, mmHg	88 ± 17	86 ± 16	87 ± 15	83 ± 15	0.149
Prior tPA	7 (25.9)	21 (45.9)	70 (41.2)	61 (46.9)	0.223
TOAST classification					
Large artery atherosclerosis	10 (37)	18 (39.1)	70 (41.2)	72 (55.4)	**0.047**
Cardioembolism	16 (59.3)	25 (54.3)	87 (51.2)	46 (35.4)	**0.013**
Other	1 (3.7)	3 (6.5)	13 (7.6)	12 (9.2)	0.875
Occlusion site					
Anterior circulation	23 (85.2)	38 (82.6)	138 (82.1)	102 (79.1)	0.826
ICA/ICA terminal bifurcation	8 (29.6)	24 (52.2)	56 (33.3)	46 (35.7)	0.102
Middle cerebral artery	15 (55.6)	14 (30.4)	82 (48.8)	56 (43.4)	0.099
Posterior circulation	4 (14.8)	8 (17.4)	30 (17.9)	27 (20.9)	0.848
Basilar artery	4 (14.8)	5 (10.9)	14 (8.3)	16 (12.4)	0.348
Vertebral artery	0	2 (4.3)	15 (8.9)	10 (7.8)	0.404
Posterior cerebral artery	0	1 (2.2)	1 (0.5)	1 (0.8)	0.618
**Laboratory tests**					
Platelet count, 10^9/L	158 (137–232)	171 (143–220)	174.5 (141–223)	189 (144–231)	0.446
PT/INR	1.04 (0.99–1.10)	1.02 (0.99–1.07)	1.01 (0.96–1.08)	0.99 (0.95–1.06)	**0.034**
Glucose, mmol/L	7.0 (5.8–9.4)	7.0 (5.6–8.2)	6.5 (5.4–8.0)	6.2 (5.1–7.5)	**0.027**
Glycosylated hemoglobin, %	5.8 (5.7–6.8)	6.2 (5.6–6.9)	5.9 (5.5–6.4)	5.8 (5.5–6.3)	0.284
Total cholesterol, mmol/L	3.54 (2.98–4.85)	3.77 (3.32–4.78)	4.14 (3.51–4.87)	4.35 (3.59–5.14)	**0.025**
LDL, mmol/L	2.21 (1.78–2.85)	2.29 (1.73–3.07)	2.43 (1.92–3.06)	2.70 (2.12–3.24)	**0.037**
HDL, mmol/L	1.13 ± 0.33	1.13 ± 0.30	1.12 ± 0.27	1.16 ± 0.31	0.667
Triglyceride, mmol/L	0.9 (0.75–1.85)	1.02 (0.73–1.61)	1.04 (0.76–1.47)	1.09 (0.75–1.57)	0.843
Urea nitrogen, mmol/L	9.64 (7.45–12.10)	7.50 (5.41–8.95)	5.93 (4.81–7.21)	4.70 (4.09–5.54)	**< 0.001**
Homocysteine, umol/L	14.45 (12.00–17.88)	14.66 (12.14–18.33)	13.01 (10.89–16.78)	11.51 (9.76–13.91)	**< 0.001**

*TIA* transient ischemic attack, *NIHSS* National Institutes of Health Stroke Scale, *OTT*, onset to treatment time, *SBP* systolic blood pressure, *DBP* diastolic blood pressure, *tPA* tissue plasminogen activator, *ICA* internal carotid artery, *PT/INR* prothrombin time and international normalized ratio, *LDL* low density lipoprotein, *HDL* high density lipoprotein

^a^Smoking status including both former and current

### Univariable analysis

In univariable analysis, as continuous variable, reduced eGFR (per 10 ml/min/1.73 m^2^ decrease) was associated with increased risk of sICH (*p* = 0.001), death in hospital (*p* = 0.01), death at 3 months (*p* < 0.001) and poor functional outcome (*p* < 0.001), but it was not associated with recanalization rate (*p* = 0.51) (Supplemental Table 1).

In unadjusted analysis, as categorical variable, compared to normal eGFR (G1), reduced eGFR was associated with increased risk of sICH (*p* = 0.021), death at 3 months (*p* = 0.008) and poor functional outcome (*p* < 0.001), but it was not associated with recanalization rate (*p* = 0.506) or death in hospital (*p* = 0.343) (Table 2).

**Table 2:** Multivariable analysis of outcomes according to the eGFR

	eGFR (ml/min/1.73 m^2^)				
	≤44 (G3b–5) *n* = 27	45–59 (G3a) *n* = 46	60–89 (G2) *n* = 170	≥90 (G1) *n* = 130	*p*-trend
**TICI 2b–3**					
N (%)	24 (88.9)	39 (84.8)	146 (85.9)	118 (90.8)	
Unadjusted OR (95% CI)	0.75 (0.19–2.88) *p* = 0.67	0.52 (0.19–1.43) *p* = 0.206	0.59 (0.28–1.26) *p* = 0.175	–	0.506
Model 1^a^ OR (95% CI)	0.88 (0.21–3.77) *p* = 0.868	0.59 (0.20–1.74) *p* = 0.337	0.68 (0.29–1.60) *p* = 0.375	–	0.747
Model 2^b^ OR (95% CI)	0.89 (0.20–3.96) *p* = 0.882	0.67 (0.21–2.07) *p* = 0.481	0.71 (0.29–1.71) *p* = 0.443	–	0.855
**sICH**					
N (%)	5 (18.5)	10 (21.7)	17 (10.0)	8 (6.2)	
Unadjusted OR (95% CI)	**3.44 (1.03–11.48)*p*** **= 0.045**	**4.32 (1.59–11.78)*p*** **= 0.004**	1.74 (0.73–4.16) *p* = 0.215	–	**0.021**
Model 1^a^ OR (95% CI)	3.27 (0.86–12.40) *p* = 0.081	**4.15 (1.41–12.24)*p*** **= 0.01**	1.67 (0.63–4.46) *p* = 0.305	–	**0.042**
Model 2^c^ OR (95% CI)	2.12 (0.48–9.35) *p* = 0.320	**4.07 (1.30–12.75)*p*** **= 0.016**	1.71 (0.61–4.83) *p* = 0.311	–	0.095
**Death, in hospital**					
N (%)	5 (18.5)	9 (19.6)	23 (13.5)	13 (10.0)	
Unadjusted OR (95% CI)	2.05 (0.66–6.32) *p* = 0.214	2.19 (0.87–5.53) *p* = 0.098	1.41 (0.68–2.90) *p* = 0.353	–	0.343
Model 1^a^ OR (95% CI)	1.73 (0.49–6.09) *p* = 0.392	1.90 (0.69–5.21) *p* = 0.213	1.25 (0.54–2.86) *p* = 0.602	–	0.604
Model 2^d^ OR (95% CI)	1.21 (0.29–5.08) *p* = 0.793	1.33 (0.42–4.19) *p* = 0.627	1.12 (0.45–2.81) *p* = 0.807		0.970
**Death, 3m**^e^					
N (%)	10 (38.5)	12 (26.7)	44 (27.0)	16 (12.9)	
Unadjusted OR (95% CI)	**4.22 (1.63–10.89)*p*** **= 0.003**	**2.46 (1.06–5.71)*p*** **= 0.037**	**2.50 (1.33–4.68)*p*** **= 0.004**	–	**0.008**
Model 1^a^ OR (95% CI)	**3.36 (1.18–9.53)*p*** **= 0.023**	2.07 (0.84–5.10) *p* = 0.114	**2.11 (1.03–4.29)*p*** **= 0.04**	–	0.107
Model 2^f^ OR (95% CI)	**3.46 (1.17–10.26)*p*** **= 0.025**	1.85 (0.71–4.80) *p* = 0.206	2.09 (0.99–4.41) *p* = 0.054	–	0.13
**mRS 3–6, 3m**^e^					
N (%)	20 (76.9)	32 (71.1)	116 (71.2)	60 (48.4)	
Unadjusted OR (95% CI)	**3.56 (1.34–9.45)*p*** **= 0.011**	**2.63 (1.26–5.47)*p*** **= 0.01**	**2.63 (1.62–4.29)*p*** **< 0.001**	**–**	**< 0.001**
Model 1^a^ OR (95% CI)	1.76 (0.61–5.07) *p* = 0.293	1.63 (0.74–3.60) *p* = 0.230	1.51 (0.85–2.67) *p* = 0.159	–	0.448
Model 2^g^ OR (95% CI)	1.47 (0.45–4.77) *p* = 0.524	1.53 (0.62–3.76) *p* = 0.353	1.48 (0.78–2.83) *p* = 0.232	–	0.644

^a^ Adjusted for age and sex

^b^ Adjusted for age, sex, NIHSS, OTT and glucose

^c^ Adjusted for age, sex, NIHSS, OTT, previous TIA/stroke, SBP and glucose

^d^ Adjusted for age, sex, NIHSS, OTT, coronary artery disease, PT/INR and glucose

^e^ 15 patients were lost to follow-up. 6 in G1, 7 in G2, 1 in G3a, 1 in G3b–5

^f^ Adjusted for age, sex, NIHSS, OTT and glucose

^g^ Adjusted for age, sex, NIHSS, OTT, atrial fibrillation, smoking, glucose and HDL

### Multivariable analysis

After adjusting for age and sex (Model 1), compared to normal eGFR (G1), reduced eGFR was associated with a higher risk of sICH (G3a, *p* = 0.01) and death at 3 months (G2, *p* = 0.04; G3b–5, *p* = 0.023) (Table 2).

In multivariable logistic regression analysis (Model 2), we adjusted all the potential confounders. Compared to normal eGFR (G1), reduced eGFR was still associated with increased risk of sICH (G3a, *p* = 0.016) and death at 3 months (G3b–5, *p* = 0.025). However, it was not associated with a higher risk of death in hospital (*p* = 0.970) or poor functional outcome (*p* = 0.644). The recanalization rate was not influenced by renal function (*p* = 0.855) (Table 2).

## Discussion

Our study, which included 373 AIS patients, evaluated the influence of renal function on outcomes after MT. The results revealed that in short-term outcomes, compared with normal eGFR (G1), reduced eGFR was not associated with recanalization rate and death in hospital. Renal dysfunction G3a (45–59 mL/min/1.73 m^2^) increased the risk of sICH. In long-term outcomes, renal dysfunction G3b-5 (≤44 ml/min/1.73 m^2^) increased the risk of death at 3 months. However, there was no appreciable effect of reduced eGFR on functional outcome at 3 months.

Several previous observational studies investigated the relationship between renal function and stroke outcomes. However, only one study included MT patients and their results were conflicting [4–8, 17–21]. Some studies found that reduced eGFR significantly increased the risk of stroke mortality [4, 5] and it was associated with poor outcome and sICH after intravenous thrombolysis [6]. However, other studies found such influence was not statistically significant [7, 8]. These discrepancies may be explained by the following reasons. First, the stroke type, some studies included both ischemic and hemorrhagic stroke [5, 7, 18, 19], while others were one type only [4, 6, 8, 17, 20, 21]. Second, different onset time. Some study included the stroke patients within 24 h of onset [6, 8, 18, 21], while others were 14 days [4] or 7 days [5]. Third, the difference of standard treatment. Some studies were conducted 10 years ago [14, 16–20]. The standard treatment recommended by guidelines were updated during this period, which may cause the different outcomes.

Unlike other AIS treatment, in MT, the risk of contrast-induced nephropathy (CIN) was increased with reduced eGFR. It was associated with increased morbidity, hospital stay and mortality [13]. There was only one previous study conducted by Laible et al. investigated the renal function and MT outcomes [21].

In short-term outcomes, our study found eGFR 45–59 ml/min/1.73 m^2^ (G3a) was associated with the risk of sICH. However, the study of Laible did not observe any relationship between renal dysfunction (eGFR < 60 ml/min/1.73 m^2^) and sICH or any ICH. In the study of Laible et al. with German population, the overall sICH rate was 6.8%. It was significantly lower than our study with Chinese population (10.7%). In another Chinese study evaluating sICH after MT, the sICH rate was even higher at about 16% [22]. Not only MT, in intravenous thrombolysis therapy, Asian population also had a significantly higher risk of sICH compared with the white group [23]. These results indicated that race/ethnic may cause the difference in bleeding risk after MT. Thus, we suggested that Chinese/Asian patients should be carefully monitored during and after MT procedure.

In long-term outcomes of 3 months mortality and poor outcome (mRS 3–6), our results were in accordance with Laible et al. In Laible et al., as a continuous variable, eGFR (each decrease of 10 ml/min/1.73 m^2^) was associated with increased risk of mortality while as categorical variable, our study showed the group of eGFR ≤44 ml/min/1.73 m^2^ (G3b–5) increased such risk. However, both our two studies observed that even though the mortality risk was increased, the functional outcome seemed not to be influenced by renal function. The plausible explanation of this result is still unidentified. But we presumed that patient with reduced eGFR had more comorbidities such as mineral and bone disorder, malnutrition and anemia. These comorbidities may not directly influence the neurological outcomes but it could increase the risk of death in elderly population.

### Limitations

There were some limitations in our study. First, our study was a single-center observational cohort with only Chinese patients, the results may not be applicable to other populations. Second, CKD-EPI equation using creatinine has been proved to be more accurate than MDRD equation and has been recommended by current guidelines [15, 24]. However, in our study, the mean age of the patient was 71.2 years. The equation combined with both creatinine and cystatin C may be more accurate than using creatinine alone for elderly individuals [25].

Despite the limitations, our study revealed that reduced eGFR did not represent a risk factor for recanalization rate, death in hospital and poor functional outcome. Renal dysfunction may not be considered as an exclusion criteria for MT.

## Conclusions

Our study suggested that in AIS patients who underwent MT, reduced eGFR was associated with increased risks of sICH and death at 3 months. However, there were no appreciable effects of reduced eGFR on recanalization rate, death in hospital and functional outcome at 3 months.

## Supplementary information

**Additional file 1: Table S1.** Univariable analysis of clinical characteristics in patients treated with EVT.

## Data Availability

All data have been presented within the manuscript and additional supporting files.

## Abbreviations

CKD: Chronic kidney disease
eGFR: Estimated glomerular filtration rate
IVT: Intravenous thrombolysis
sICH: Symptomatic intracerebral hemorrhage
MT: Mechanical thrombectomy
CM: Iodinated contrast media
AKI: Acute kidney injury
PT/INR: Prothrombin time and international normalized ratio
MRI: Magnetic resonance imaging
CT: Computerized tomographic scan
CKD-EPI: CKD Epidemiology Collaboration Equation
TICI: Thrombolysis in Cerebral Infarction Scale
NIHSS: National Institutes of Health Stroke Scale
mRS: Modified Rankin Scale
SD: Standard deviation
IQR: Inter-quartile range
CIN: Contrast-induced nephropathy
