# Supplementary material for: Influence of renal function on stroke outcome after mechanical thrombectomy: a prospective cohort study
Source: BMC Neurol. 2020 Apr 14;20:134. doi: 10.1186/s12883-020-01720-5 (PMC7155325; doi:10.1186/s12883-020-01720-5)
Supplement: Supplementary file 1 — Additional file 1: Table S1. Univariable analysis of clinical characteristics in patients treated with EVT. [file 12883_2020_1720_MOESM1_ESM.doc]

**Supplemental Table 1 Univariable analysis of clinical characteristics in patients treated with EVT**

|  | **TICI 2b–3** | | **sICH** | | **Death, in hospital** | | **Death, 3m^a^** | | **mRS 3–6^a^** | |
| --- | --- | --- | --- | --- | --- | --- | --- | --- | --- | --- |
|  | OR (95%CI) | *p* | OR (95%CI) | *p* | OR (95%CI) | *p* | OR (95%CI) | *p* | OR (95%CI) | *p* |
| **Demographic** |  |  |  |  |  |  |  |  |  |  |
| Age, y | 0.98 (0.95–1.01) | 0.2 | 1.02 (0.99–1.05) | 0.21 | 1.02 (0.99–1.04) | 0.26 | **1.03 (1.01–1.06)** | **0.01** | **1.06 (1.04–1.08)** | **< 0.001** |
| Male | 1.20 (0.63–2.27) | 0.59 | 1.11 (0.56–2.21) | 0.77 | **2.03 (1.02–4.02)** | **0.04** | 1.23 (0.73–2.97) | 0.43 | **0.57 (0.36–0.90)** | **0.02** |
| **Cardiovascular risk factors** |  |  |  |  |  |  |  |  |  |  |
| Hypertension | 0.55 (0.25–1.23) | 0.15 | 1.53 (0.68–3.44) | 0.31 | 1.39 (0.68–2.83) | 0.36 | 1.28 (0.72–2.28) | 0.40 | 1.14 (0.70–1.85) | 0.60 |
| Diabetes | **0.48 (0.24–0.96)** | **0.04** | 1.17 (0.53–2.57) | 0.70 | 1.71 (0.87–3.36) | 0.12 | 0.88 (0.47–1.66) | 0.69 | 1.46 (0.83–2.56) | 0.19 |
| Coronary artery disease | 0.99 (0.47–2.11) | 0.99 | 0.69 (0.29–1.61) | 0.39 | **0.43 (0.18–1.04)** | **0.06** | 0.64 (0.34–1.22) | 0.17 | 1.18 (0.70–2.00) | 0.53 |
| Atrial fibrillation | 0.91 (0.47–1.76) | 0.78 | 1.18 (0.60–2.33) | 0.64 | 1.46 (0.80–2.68) | 0.22 | 1.12 (0.67–1.87) | 0.67 | **1.56 (0.97–2.47)** | **0.07** |
| TIA/stroke | 0.76 (0.38–1.53) | 0.45 | **0.31 (0.11–0.89)** | **0.03** | 0.54 (0.24–1.20) | 0.13 | 0.67 (0.37–1.23) | 0.20 | 1.03 (0.62–1.69) | 0.92 |
| Smoking | 1.35 (0.68–2.69) | 0.39 | 0.77 (0.38–1.58) | 0.48 | 0.84 (0.44–1.58) | 0.58 | 0.74 (0.44–1.26) | 0.27 | **0.52 (0.33–0.81)** | **0.004** |
| **Clinical features on admission** |  |  |  |  |  |  |  |  |  |  |
| NIHSS score | **0.94 (0.90–0.97)** | **0.001** | **1.06 (1.01–1.10)** | **0.01** | **1.08 (1.04–1.12)** | **< 0.001** | **1.08 (1.04–1.11)** | **< 0.001** | **1.14 (1.09–1.18)** | **< 0.001** |
| OTT, min | 1.00 (1.00–1.00) | 0.29 | 1.00 (1.00–1.00) | 0.78 | 1.00 (1.00–1.00) | 0.99 | 1.00 (1.00–1.00) | 0.11 | 1.00 (1.00–1.00) | 0.46 |
| SBP, mmHg | 1.00 (0.99–1.01) | 0.82 | 1.01 (1.00–1.03) | 0.14 | 1.00 (0.99–1.01) | 0.80 | 1.01 (1.00–1.02) | 0.33 | 1.01 (1.00–1.01) | 0.30 |
| DBP, mmHg | 1.01 (0.99–1.04) | 0.22 | 1.00 (0.98–1.02) | 0.93 | 1.00 (0.98–1.02) | 0.74 | 1.00 (0.99–1.02) | 0.61 | 1.01 (0.99–1.02) | 0.27 |
| Prior tPA | 1.34 (0.70–2.58) | 0.38 | 0.86 (0.44–1.69) | 0.67 | 1.17 (0.64–2.13) | 0.61 | 0.77 (0.46–1.27) | 0.30 | 0.77 (0.50–1.18) | 0.23 |
| **Laboratory tests** |  |  |  |  |  |  |  |  |  |  |
| Creatinine, umol/l | 1.00 (0.99–1.01) | 0.81 | **1.02 (1.01–1.03)** | **0.001** | **1.02 (1.01–1.03)** | **0.002** | **1.02 (1.01–1.02)** | **< 0.001** | **1.01 (1.00–1.02)** | **0.03** |
| eGFR (per 10 ml/min/1.73 m^2^ decrease) | 0.95 (0.82–1.10) | 0.51 | **1.28 (1.10–1.50)** | **0.001** | **1.20 (1.05–1.38)** | **0.01** | **1.26 (1.12–1.41)** | **< 0.001** | **1.29 (1.15–1.45)** | **< 0.001** |
| Platelet count, 10^9/L | 1.00 (0.99–1.00) | 1.00 | 1.00 (0.99–1.00) | 0.26 | 1.00 (1.00–1.01) | 0.36 | 1.00 (1.00–1.00) | 0.98 | 1.00 (1.00–1.00) | 0.61 |
| PT/INR | 1.29 (0.16–10.15) | 0.81 | 1.73 (0.25–12.10) | 0.58 | **4.93 (1.12–21.62)** | **0.04** | 2.59 (0.66–10.16) | 0.17 | 1.64 (0.41–6.49) | 0.48 |
| Glucose, mmol/L | **0.90 (0.80–1.00)** | **0.06** | **1.15 (1.03–1.29)** | **0.02** | **1.18 (1.06–1.31)** | **0.002** | **1.17 (1.07–1.29)** | **0.001** | **1.31 (1.16–1.48)** | **< 0.001** |
| Total cholesterol, mmol/L | 0.84 (0.64–1.10) | 0.20 | 0.90 (0.66–1.23) | 0.51 | 1.13 (0.87–1.46) | 0.35 | 1.06 (0.85–1.32) | 0.60 | 1.09 (0.89–1.32) | 0.40 |
| LDL, mmol/L | 0.82 (0.60–1.11) | 0.19 | 0.82 (0.56–1.20) | 0.30 | 1.20 (0.90–1.62) | 0.22 | 1.12 (0.87–1.44) | 0.39 | 1.06 (0.84–1.33) | 0.62 |
| HDL, mmol/L | 1.45 (0.46–4.52) | 0.53 | 1.14 (0.36–3.57) | 0.83 | 1.00 (0.35–2.89) | 0.99 | 0.80 (0.33–1.91) | 0.61 | **2.39 (1.09–5.24)** | **0.03** |
| Homocysteine, umol/L | 1.02 (0.97–1.06) | 0.53 | 1.00 (0.95–1.04) | 0.91 | 0.98 (0.94–1.03) | 0.44 | 0.98 (0.95–1.02) | 0.31 | 0.99 (0.97–1.02) | 0.60 |

TICI, thrombolysis in cerebral infarction scale; sICH, symptomatic intracranial hemorrhage; mRS, modified Rankin Scale; OR, odds ratio; CI, confidence interval; TIA, transient ischemic attack; NIHSS, National Institutes of Health Stroke Scale; OTT, onset to treatment time; SBP, systolic blood pressure; DBP, diastolic blood pressure; tPA, tissue plasminogen activator; PT/INR, prothrombin time and international normalized ratio; LDL, low density lipoprotein; HDL, high density lipoprotein.

^a^15 patients were lost to follow-up.
